# Endogenous Activation of mGlu5 Metabotropic Glutamate Receptors Is Required for Oligodendrocyte Maturation and Myelinogenesis in Mice

**DOI:** 10.3390/cells15141311

**Published:** 2026-07-22

**Authors:** Roxana Paula Ginerete, Roberta Facchinetti, Alessia Ceccherelli, Giada Mascio, Sara Rubio Casado, Luisa Di Menna, Sonia Castaldi, Matteo Caridi, Valeria Bruno, Ferdinando Nicoletti, Giuseppe Battaglia, Caterina Scuderi, Rosamaria Orlando

**Affiliations:** 1 Department of Molecular Pathology, IRCCS Neuromed, 86077 Pozzilli, Italy; roxana.ginerete@neuromed.it (R.P.G.); giada.mascio@neuromed.it (G.M.); luisa.dimenna@neuromed.it (L.D.M.); sonia.castaldi@neuromed.it (S.C.); valeria.bruno@uniroma1.it (V.B.); ferdinando.nicoletti@uniroma1.it (F.N.); rosamaria.orlando@neuromed.it (R.O.); 2 Department of Life Science, Health, and Health Professions, Link Campus University, 00165 Roma, Italy; r.facchinetti@unilink.it; 3 Department of Physiology and Pharmacology “V. Erspamer”, Sapienza University, 00185 Roma, Italy; alessia.ceccherelli@uniroma1.it (A.C.); matteo.caridi@uniroma1.it (M.C.); caterina.scuderi@uniroma1.it (C.S.); 4 Department of Immunology, Ophthalmology and ORL, University Complutense, 28040 Madrid, Spain; srubio02@ucm.es

**Keywords:** myelin, mGlu5 receptor, oligodendrocytes, neurodevelopment, MTEP

## Abstract

mGlu5 metabotropic glutamate receptors are highly expressed and functional in early postnatal life, but their role in brain development remains to be elucidated. We now report that mGlu5^−/−^ mice showed a large reduction in mRNA and protein levels of myelin-associated glycoprotein (MAG), myelin oligodendrocyte glycoprotein (MOG), and myelin basic protein (MBP) in the cerebral cortex and cerebellum at postnatal day (PND) 14. Expression of these proteins reflects oligodendrocyte differentiation and myelin formation in the CNS, a process that is prominent in the second week of postnatal life in mice. Expression of myelin markers recovered at later developmental stages, with the exception of MAG and MBP levels in the cerebellum, which were still reduced in mGlu5^−/−^ mice. We also observed a large reduction in myelin protein markers in the cerebral cortex and cerebellum of mice treated with the mGlu5 receptor negative allosteric modulator MTEP (3 mg/kg, i.p.) from PND 7 to PND 14. MTEP-treated mice also showed reduced motor coordination and sensorimotor function at PND 14. Treatment with MTEP reduced oligodendrocyte maturation without affecting proliferation of oligodendrocyte progenitor cells in primary cultures prepared from the rat cerebral cortex. These findings suggest that endogenous activation of mGlu5 receptors contributes to myelin formation during postnatal development in mice.

## 1. Introduction

Myelin dysfunction is associated with neurodevelopmental disorders, including autism spectrum disorders, and correlates with cognitive impairment and intellectual disability [1]. Hence, unravelling the mechanisms underlying oligodendrocyte maturation and myelin formation may disclose new targets for therapeutic intervention [1]. Oligodendrocytes originate from oligodendrocyte progenitor cells (OPC), which proliferate and migrate in the CNS at specific timepoints known as “waves” [2,3]. The progression through the oligodendrocyte lineage is temporally regulated by a combination of transcription factors and surface antigens [4,5]. Highly migratory and proliferating OPCs are identified by the expression of specific markers, including the platelet-derived growth factor receptor-α (PDGFRα). PDGFRα regulates the timing of OPC differentiation and is downregulated when OPCs begin to differentiate [6,7]. OPCs differentiate into premyelinating (immature) oligodendrocytes and, finally, into mature myelinating oligodendrocytes, which are identified by the expression of myelin basic protein (MBP), myelin-associated glycoprotein (MAG), myelin oligodendrocyte glycoprotein (MOG), the enzyme 2′,3′-cyclic-nucleotide 3′-phosphodiesterase (CNPase), and proteolipid protein (PLP) [8,9].

Metabotropic glutamate receptors subtype 5 (mGlu5) are coupled to G_q/11_, and their activation stimulates polyphosphoinositide (PI) hydrolysis with ensuing formation of inositol-1,4,5-trisphosphate (InsP_3_) and diacylglycerol (DAG). InsP_3_ releases Ca^2+^ from intracellular stores, whereas DAG activates protein kinase C (PKC) [10]. mGlu5 receptors are highly expressed in brain tissue during the first two weeks of postnatal life [11,12,13,14,15], when glutamate stimulates PI hydrolysis to a larger extent than in adult life [16]. This developmental age coincides with the onset of myelination, which, in the rodent brain, begins in the first week after birth [8,17,18,19,20]. OPCs express the mGlu5a receptor (one of the two splice variants of mGlu5 receptors) [21]. mGlu5 receptor expression is prominent in the early phase of OPC differentiation in culture, and the expression progressively declines during oligodendrocyte maturation [22]. In addition, at postnatal day 6 (PND 6), premyelinating oligodendrocytes express mGlu5 receptors to a greater extent than neurons, astrocytes, and microglia, and pharmacological activation of mGlu5 receptors attenuates the loss of white matter MBP caused by neonatal hypoxia/ischemia in a rat model of perinatal periventricular leukomalacia [23]. The link between mGlu5 receptors and myelin formation is strengthened by the evidence that, in zebrafish, pharmacological inhibition and loss-of-function mutations of mGlu5 receptors impair myelin growth, whereas mGlu5 receptor activation promotes myelin sheath elongation [24].

Here we examined the role of mGlu5 receptors in oligodendrocyte differentiation, measuring the expression of myelin markers in the intact brain and in primary cultures of OPC/oligodendrocytes.

## 2. Materials and Methods

### 2.1. Materials

3-((2-methyl-4-thiazolyl)ethynyl) pyridine (MTEP) was purchased from Tocris Bioscience (Bristol, UK).

### 2.2. Animals

All experimental procedures were performed in conformity with the Italian (D.L. 26/2014) and European Union Directive (2010/63/EU) on the protection of animals used for scientific purposes. The study was approved by the local Organism for Animal Welfare (OPBA) of Neuromed Institute and by the Italian Ministry of Health (authorization number: 82/2022-PR). Immunohistochemical analysis was performed in tissue collected from mice used in another project (authorization number: 1135/2020-PR). Primary OPC cultures were performed in accordance with authorization number 313/2024-PR. mGlu5 receptor knockout (mGlu5^−/−^) mice were originally purchased from Jackson Laboratory, Bar Harbor, ME (B6;129-Grm5tm1Rod/J; Stock No: 003558). Wild-type and mGlu5^−/−^ mice were generated by heterozygous mating. For experiments, mice of the two genotypes were generated by homozygous breeding. Genotyping was carried out by PCR analysis according to the instructions of Jackson Laboratory. All mice were housed in a controlled temperature room (21–23 °C, humidity 40–50%) and maintained on a 12-h light/dark cycle with food and water ad libitum. All efforts were made to minimize the number of animals and to alleviate their discomfort. We used mGlu5^−/−^ and wild-type mice of both sexes for in vivo studies, and Sprague-Dawley pups (2 days old) for in vitro studies.

### 2.3. Experimental Design

#### 2.3.1. In Vivo Experiments

mGlu5^−/−^ and wild-type mice at PND 14, 21, and 30 from 3 different litters were used for behavioral analyses (*n* = 8), immunoblot (*n* = 6), and RT-PCR analysis (*n* = 5–7) of myelin markers in the cerebral cortex and cerebellum. Mice at PND 4 were used for immunoblot (*n* = 1) and RT-PCR analysis (*n* = 5–6), whereas mice at PND 7 were used exclusively for RT-PCR analysis (*n* = 4–8). Immunohistochemical analysis was performed in brain and cerebellar samples collected from another set of experiments in mice at PND 14 (*n* = 3). In another set of experiments, wild-type mice were treated i.p. with either saline or MTEP (3 mg/kg) once a day from PND 7 to PND 14. Two hours after the last injection, mice were subjected to behavioral analyses (*n* = 6) and then killed for immunoblot analysis of myelin protein markers (*n* = 5–6). Separate groups of MTEP- or saline-injected mice were used for immunohistochemical analysis (*n* = 3). We used a total number of 96 mice.

#### 2.3.2. OPCs and Oligodendrocytes Primary Cell Cultures

Primary OPCs were isolated from the cerebral cortices of male and female pups (postnatal days 0–2, total *n* = 18 pups). Cells were seeded into 75 cm^2^ tissue culture flasks at a density of one pup per flask. Cultures were maintained at 37 °C in a humidified atmosphere containing 5% CO_2_, and the medium was replaced after 3 days. Ten days after isolation, microglial cells were removed by shaking the flasks for 1 h at 37 °C. OPCs were separated from astrocytes by overnight shaking at 37 °C. OPCs were plated either onto poly-D-lysine hydrobromide-coated (70,000–150,000 MW; Sigma-Aldrich, Milan, Italy) 24-well plates for Western blot analysis or onto coated coverslips in 96-well plates for immunofluorescence studies. For proliferation experiments, cells were cultured for 2 days in vitro (DIV) for Western blot and for 2 or 3 DIV for immunofluorescence in DMEM supplemented with PDGF-AA 10 ng/mL, biotin 10 ng/mL, hydrocortisone 20 nM, 100 U/mL penicillin and 100 μg/mL streptomycin (Sigma-Aldrich), bFGF 10 ng/mL, bovine serum albumin 1%, and 2% N2 supplement (ThermoFisher Scientific, Waltham, MA, USA). For differentiation experiments, cells were cultured for 7 DIV for Western blot and from 2 to 6 DIV for immunofluorescence in DMEM supplemented with 400 ng/mL 3,3′,5-triiodo-L-thyronine, 400 ng/mL L-thyroxine, 100 U/mL penicillin and 100 μg/mL streptomycin (Sigma-Aldrich), and 2% N2 supplement (ThermoFisher Scientific).

#### 2.3.3. Treatments

MTEP, the mGlu5 receptor negative allosteric modulator (NAM), was dissolved in saline and injected i.p. at the dose of 3 mg/kg once a day for 7 days from PND 7 to PND 14. This dose was selected based on previous studies showing >75% mGlu5 receptor occupancy in the CNS after i.p. administration of 3 mg/kg MTEP [25]. Primary cells cultured in proliferation medium were treated for 24 or 48 h with MTEP (1, 3, or 10 µM) or water. For differentiation experiments, cells were cultured in differentiation medium, and MTEP (1 µM) or water was added after 24 h (1 DIV) and renewed every 24 h for the next 4 days. Thus, cultures were exposed to MTEP from 1 to 5 DIV, and maturation was assessed at 2, 3, 4, 5, and 6 DIV. The toxicity of MTEP (1 µM) was measured as lactate dehydrogenase (LDH) release on the conditioned media of oligodendrocytes at the different five timepoints of observation, using the Lactate Dehydrogenase Activity Assay Kit (MAK066) (Sigma-Aldrich) following the manufacturer’s instructions.

### 2.4. Western Blot Analysis

Mice were killed by decapitation, and cerebral cortex and cerebellum were quickly dissected out, flash frozen in liquid nitrogen, and stored at −80 °C. Tissue was homogenized by sonication at 4 °C in ice-cold Triton X-lysis buffer (10 mM Tris-HCl, pH 7.4, 150 mM NaCl, 1% Triton X-100, 1 mM EDTA, pH 8, 10% glycerol, with complete Protease Inhibitor Cocktail and PhosSTOP (Roche, Meylan, France)). After centrifugation for 30 min at 13,000× *g* at 4 °C, 10 μg of proteins were resuspended in sodium dodecyl sulfate (SDS)-bromophenol blue reducing buffer containing 5% 2-mercaptoethanol and boiled before loading. Samples were separated by SDS-PAGE electrophoresis on 4–12% polyacrylamide Mini-PROTEAN TGX Precast Protein Gels and then transferred to PVDF membranes using a Trans-Blot Turbo Mini (Bio-Rad, Hercules, CA, USA). Blots were preincubated in a blocking solution of 5% non-fat dry milk (Bio-Rad) in 0.1% TBST (0.1 M Tris base, 0.1% Tween 20, pH 7.4) for 1 h at room temperature, incubated with primary antibodies overnight at 4 °C, and, after washing, with a horseradish peroxidase-conjugated anti-rabbit or anti-mouse antibody (1:5000). Immunostaining was revealed by the enhanced ECL Western blotting analysis system (ThermoFisher Scientific) and by the Chemidoc computerized densitometer (Bio-Rad), quantified by ImageLab 3.0 software (Bio-Rad). The same procedure was used to extract and resolve proteins from oligodendrocyte cell cultures.

Primary antibodies: rabbit polyclonal anti-MBP (Abcam, Cambridge, UK, code: ab40390, 1:1000); mouse monoclonal anti-MAG (Santa Cruz Biotechnology, Dallas, TX, USA, code: sc-166849, 1:1000); mouse monoclonal anti-MOG (Santa Cruz, code: 166172, 1:1000); rabbit anti-CNPase (AbClonal, Düsseldorf, Germany, code: A1018, 1:1000); mouse-anti-PDGFRα (Novus Biologicals, Centennial, CO, USA, code: AF1062-SP, 1:1000); or mouse monoclonal anti-β-actin (Sigma-Aldrich, code: A5441, 1:50,000).

### 2.5. Quantitative Real-Time PCR

Tissue was placed in Eppendorf tubes, flash frozen in liquid nitrogen, and stored at −80 °C until RNA extraction. Total RNA was isolated using Trizol/chloroform according to the manufacturer’s protocol (Invitrogen, Carlsbad, CA, USA). After DNase treatment for 10 min at room temperature (Qiagen, Hilden, Germany), single strand cDNA was synthesized starting from 1.0 or 2.0 μg of total RNA using Superscript III (Invitrogen) and random hexamers. Real-time PCR was performed on 15 ng of cDNA by using specific primers and Power SYBR Green Master Mix (Bio-Rad) on an Applied Biosystems Step-One instrument. Thermal cycler conditions were as follows: 10 min at 95 °C, 40 cycles of denaturation (15 s at 95 °C), and combined annealing/extension (1 min at 58–60 °C). For relative quantification of gene expression, the comparative threshold cycle (ΔΔCt) method was used. Ct values were calculated for each gene and normalized to glyceraldehyde-3-phosphate dehydrogenase (*Gapdh*). Each sample was analyzed in duplicate. PCR product specificity was validated through melting curve analysis.

Primer sequences used for real-time PCR analysis: *Mbp* (forward, 5′-CTGGCCACAGCAAGTACCAT; reverse, 3′-CCCCTGTCACCGCTAAAGAA), *Mag* (forward, 5′-GCGTTCCTCAGCTCCTCAT; reverse, 3′-CCTCAGAGAGAATCGGCCC), *Mog* (forward, 5′-ATATCTGGCAAGGGTGACGTG; reverse, 3′-GCCCAACCTCCATGTAAGCA), *Pdgfrα* (forward, 5′-CCTCAGAGAGAATCGGCCC; reverse, 3′-CCATAGCTCCTGAGACCCGC), *Gapdh* (forward, 5′-CGTCCCGTAGACAAAATGGT; reverse, 3′-TCAATGAAGGGGTCGTTGAT).

### 2.6. Immunofluorescent Staining

Mice were anesthetized and subjected to transcardial perfusion with saline followed by ice-cold 4% paraformaldehyde (Santa Cruz) in phosphate buffer-saline, pH 7.4. Brains were removed, post-fixed overnight in the same fixative, and then placed in 30% sucrose/0.1 M phosphate buffer for at least 24 h. Coronal sections (30 μm) were cut on a freezing microtome and stored in cryoprotective solution at −20 °C. Frozen sections were blocked with 5% normal horse serum at room temperature for 2 h and then incubated with primary anti-MBP antibody (Abcam, code: ab40390, 1:200) overnight at 4 °C. After washing, sections were incubated with biotinylated anti-rabbit secondary antibody and then marked with Alexa Fluor 488 (1:200, Invitrogen) for 2 h at room temperature. Finally, sections were mounted with an anti-fading agent (Vector, Burlingame, CA, USA) and examined with Zeiss Carl Axiophot2 microscope (Zeiss, Göttingen, Germany) and processed with NIS-elements F3.0.

Oligodendrocytes in 96-well plates were fixed in 4% paraformaldehyde in phosphate buffer-saline for 20 min at room temperature. After cell permeabilization, cells were submerged for 2 h in a blocking solution containing 5% bovine serum albumin and 0.25% Triton X-100 in phosphate buffer-saline and subsequently incubated overnight at 4 °C with the blocking solution supplemented with the following primary antibodies: rabbit anti-Olig2 (Santa Cruz, code: sc-48817, 1:500), mouse anti-MBP (Santa Cruz, code: sc-271524, 1:1000), mouse anti-Ki67 (Antibodies, Stockholm, Sweden, code: A270555, 1:1000). The following day, cells were incubated for 1 h with a fresh blocking solution containing the fluorescein isothiocyanate-conjugated goat anti-rabbit IgG antibody (1:200) and tetramethylrhodamine-conjugated goat anti-mouse IgG antibody (1:200) (Jackson ImmunoResearch, Suffolk, UK). Nuclei were stained with 4,6-diamidino-2-phenylindole (DAPI, ThermoFisher Scientific, code: 62248, 1:500). Lastly, cells were covered with Fluoromount aqueous mounting medium (Sigma-Aldrich) and a glass coverslip. Data were collected using an Eclipse E600 microscope (Nikon Instruments, Tokyo, Japan) and controlled by the software NIS-Elements-BR 3.2 64 bit (Nikon Instruments). Parameters of gain and exposure were kept constant to allow comparisons between samples within the same experiment. Cell purity was assessed by using anti-NeuN antibody for neurons (Abcam, code: ab104224), anti-Iba1 antibody for microglia (Wako, Tokyo, Japan, code: 019-19741), and anti-GFAP antibody for astrocytes (Immunological Science, Rome, Italy, code: MMAB-12029), together with anti-Olig2. The culture was 92% pure with 8% of GFAP^+^ astrocytes. For cell count analysis (Ki67^+^Olig2^+^/Olig2^+^ OPCs and MBP^+^Olig2^+^/Olig2^+^ oligodendrocytes), nine random fields per condition, for each independent culture, were acquired at 20× and cells were manually counted using Fiji software, v. 2.16.0/1.54p. For protein expression and morphological analysis of MBP^+^ oligodendrocytes, 20 single cells per condition, for each independent culture, were acquired at 40×. Protein expression was analyzed by measuring the mean gray value of the signal of the single cell, subtracting the mean gray value of the background. The area occupied by MBP^+^ signal, expressed in µm^2^, was determined by drawing the perimeter of each single cell. The complexity of the MBP^+^ oligodendrocytes was assessed using the Sholl analysis plugin of Fiji software. Fluorescent images were converted into binary 8-bit images. The starting radius was set at 10 μm and step size at 5 μm, thus creating five concentric radii up to 30 µm distance from the soma. The number of cell processes intersecting these circles was plotted as a function of the radial distance from the cell soma. Several additional descriptors of cell arborization were analyzed, which were the sum of intersections (calculated as the sum of intersections detected at each concentric circle), the mean of intersections (calculated as the sum of intersections divided by intersecting radii), and the maximum intersections (calculated as the highest number of processes/branches in each cell arbor). Images were analyzed by an investigator blinded to treatments.

### 2.7. Behavioral Analyses

#### 2.7.1. Negative Geotaxis Test

Negative geotaxis was used as an automatic vestibular response to detect geogravitational stimuli and to measure sensorimotor ability in wild-type and mGlu5^−/−^ mice at PND 14, and in mice treated with either MTEP or saline at the same age. Mice were individually placed on an inclined (45°) surface with the head facing downward, and the latency to rotate 180° was recorded with a maximum of 60 s.

#### 2.7.2. Rotarod Test

The rotarod test was used for the analysis of motor coordination in wild-type and mGlu5^−/−^ mice at PND 14, PND 21, and PND 30, and in mice treated with either saline or MTEP at PND 14. At PND 14, mice were tested for 4 min with 5 min recovery intervals, and the initial rotation speed was set to 1 revolution per min (rpm), with an acceleration to 10 rpm within 4 min. This rotarod protocol was applied to PND 14 mice to account for the developmental progression of motor coordination and physical performance as described by Muttathukunnel et al. [26]. At PND 21 and PND 30, mice were tested for 5 min with 5 min recovery intervals. The initial rotation speed was 4 rpm with acceleration to 45 rpm within 5 min. Each mouse was tested for a total of three trials, and the latency to fall off the rotarod was recorded.

### 2.8. Statistical Analysis

In all graphs, values are means ± S.E.M. For immunoblot and RT-PCR data, statistical analysis was performed by Student’s *t*-test or Mann–Whitney test after Shapiro–Wilk normality test. Analysis of rotarod data was performed by two-way ANOVA for repeated measures followed by Fisher’s LSD. Data were considered significant with a *p*-value < 0.05. For in vitro experiments, analysis of proliferation (cell count) was performed by one-way ANOVA, while analysis of differentiation (cell count), MBP protein expression, and surface were performed by Student’s *t*-test after Shapiro–Wilk normality test. Sholl analysis performed on MBP^+^ cells was analyzed by two-way ANOVA followed by Sidak’s multiple comparison test, using number of intersections and distance from the soma as variables. The number of independent cultures used for each experiment is indicated by the number of dots in the graphs. We used the Grubbs’ test for the identification of outlier values.

## 3. Results

### 3.1. Age-Dependent Changes in the Expression of Myelin/Oligodendrocyte Markers in mGlu5^−/−^ Mice

**Figure 1:**
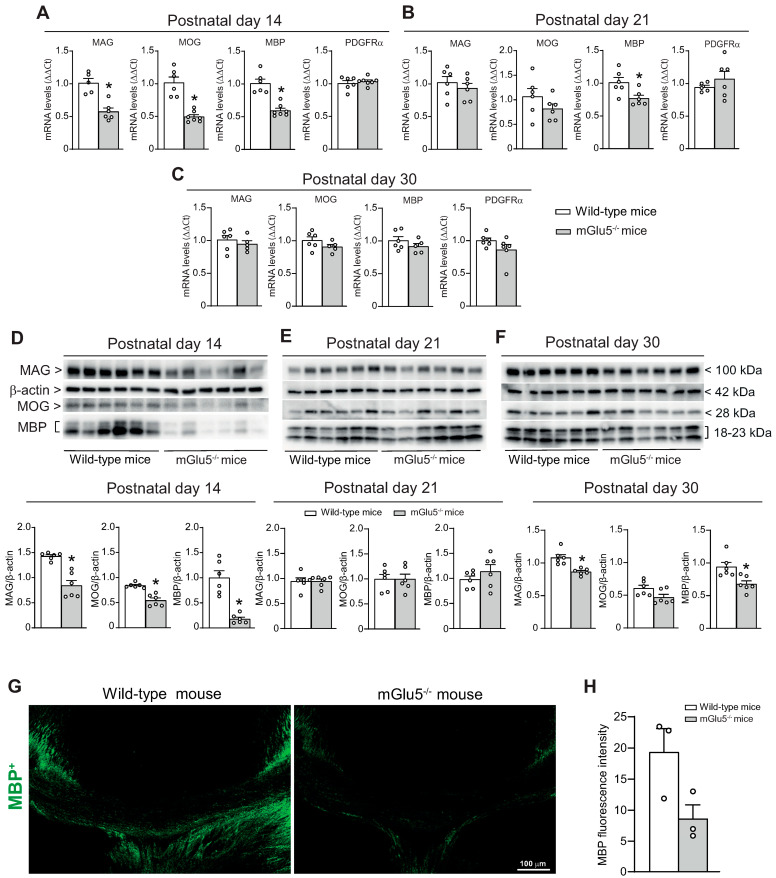
Expression of myelin and oligodendrocyte markers in the cerebral cortex of wild-type and mGlu5^−/−^ mice at PND 14, PND 21, and PND 30. (**A**–**C**) Transcript levels of MAG, MOG, MBP, and PDGFRα in the cerebral cortex of wild-type and mGlu5^−/−^ mice. Values are means ± S.E.M. of five to seven mice per group at PND 14 (**A**), PND 21 (**B**), and PND 30 (**C**). * Statistically significant vs. the corresponding values of wild-type mice (Student’s *t*-test). MAG, t_9_ = 5.004, *p* = 0.0007; MOG, t_11_ = 6.149, *p* < 0.0001; MBP, t_11_ = 5.895, *p* = 0.0001; PDGFRa, t_11_ = 0.678, *p* = 0.5118 in (**A**); MAG, t_10_ = 0.758, *p* = 0.4659; MOG, t_10_ = 1.332, *p* = 0.2125; MBP, t_10_ = 2.789, *p* = 0.0191; PDGFRa, t_9_ = 0.9688, *p* = 0.358 in (**B**); MAG, t_9_ = 0.7552, *p* = 0.4695; MOG, t_9_ = 1.473, *p* = 0.1749; MBP, t_9_ = 1.197, *p* = 0.2617; PDGFRa, t_9_ = 1.274, *p* = 0.2345 in (**C**). One value in the wild-type group for PDGFRα transcript levels in (**B**) was excluded from the analysis because the real-time PCR threshold cycle fell outside the predefined limits. (**D**–**F**) Protein levels of MAG, MOG, and MBP in the cerebral cortex of wild-type and mGlu5^−/−^ mice. Values are means + S.E.M. of six mice per group at PND 14 (**D**), PND 21 (**E**), and PND 30 (**F**). * Statistically significant vs. the corresponding values of wild-type mice (Student’s *t*-test). MAG, t_10_ = 5.542, *p* = 0.0002; MOG, t_10_ = 5.838, *p* = 0.0002; MBP, t_10_ = 5.889, *p* = 0.0002 in (**D**); MAG, U = 14, *p* = 0.5628; MOG, t_10_ = 0, *p* > 0.999; MBP, t_10_ = 1.092, *p* = 0.3003 in (**E**); MAG, t_10_ = 4.281, *p* = 0.0016; MOG, U = 6, *p* = 0.0584; MBP, t_10_ = 3.125, *p* = 0.0108 in (**F**). (**G**) Representative images of immunofluorescent staining of MBP in medial corpus callosum of wild-type and mGlu5^−/−^ mice at PND 14. Densitometric analysis is reported in (**H**), where values are means + S.E.M. of three mice per group. Student’s *t*-test, t_4_ = 2.462; *p* = 0.0695.

**Figure 2:**
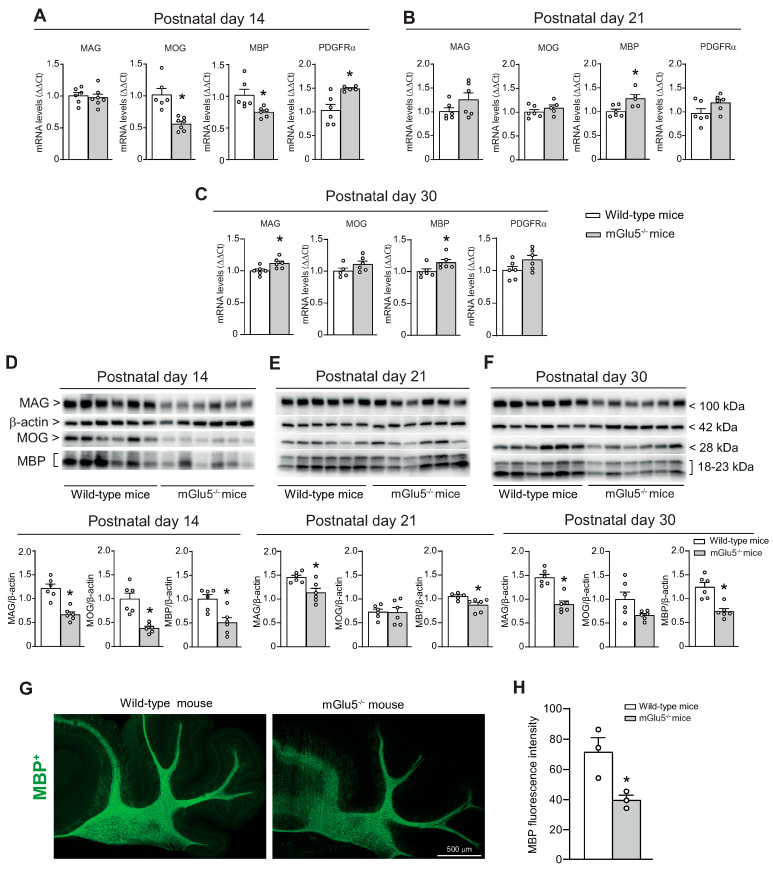
Expression of myelin and oligodendrocyte markers in the cerebellum of wild-type and mGlu5^−/−^ mice at PND 14, PND 21, and PND 30. (**A**–**C**) Transcript levels of MAG, MOG, MBP, and PDGFRα in the cerebellum of wild-type and mGlu5^−/−^ mice. Values are means ± S.E.M. of five to seven mice per group at PND 14 (**A**), PND 21 (**B**), and PND 30 (**C**). * Statistically significant vs. the corresponding values of wild-type mice (Student’s *t*-test for normally distributed values or Mann–Whitney test for non-normally distributed values after Shapiro–Wilk normality test). MAG, t_11_ = 0.3852, *p* = 0.7074; MOG, t_11_ = 4.693, *p* = 0.0007; MBP, U = 1, *p* = 0.0043; PDGFRα, t_9_ = 3.303, *p* = 0.0092 in (**A**); MAG, t_10_ = 1.529, *p* = 0.1572; MOG, t_9_ = 1.067, *p* = 0.3139; MBP, t_9_ = 2.99, *p* = 0.0152; PDGFRα, t_10_ = 1.825, *p* = 0.098 in (**B**), and MAG, t_10_ = 2.74, *p* = 0.0208; MOG, t_9_ = 1.652, *p* = 0.133; MBP, t_10_ = 2.429, *p* = 0.0355; PDGFRα, t_10_ = 1.896, *p* = 0.0872 in (**C**). One value of MBP transcript levels and two values of PDGFRα transcript levels, respectively, from the mGlu5^−/−^ group in (**A**), and one value from the wild-type group of MOG transcript levels in (**C**) were excluded from the analysis because the real-time PCR threshold cycle fell outside the predefined limits. One statistically significant outlier value was identified by Grubbs’ test in the mGlu5^−/−^ groups of MOG and MBP transcript levels in (**B**) and excluded from the analysis. (**D**–**F**) Protein levels of MAG, MOG, and MBP in the cerebellum of wild-type and mGlu5^−/−^ mice. Values are means ± S.E.M. of six mice per group at PND 14 (**D**) and PND 30 (**F**), and five to six mice per group at PND 21 (**E**). One statistically significant outlier value was identified by Grubbs’ test in the wild-type group of MBP protein levels in (**E**) and excluded from the analysis. * Statistically significant vs. the corresponding values of wild-type mice (Student’s *t*-test for normally distributed values or Mann–Whitney test for non-normally distributed values after Shapiro–Wilk normality test). MAG, t_10_ = 5.411, *p* = 0.0003; MOG, t_10_ = 4.793, *p* = 0.0007; MBP, t_10_ = 3.669, *p* = 0.0043 in (**D**); MAG, t_10_ = 3.231, *p* = 0.009; MOG, t_10_ = 0.0862, *p* = 0.9329; MBP, U = 2, *p* = 0.0152 in (**E**); MAG, t_10_ = 5.79, *p* = 0.0002; MOG, t_10_ = 2.068, *p* = 0.0655; MBP, t_10_ = 4.64, *p* = 0.0009 in (**F**). (**G**) Representative images of immunofluorescent staining of MBP in the cerebellum of wild-type and mGlu5^−/−^ mice at PND 14. Densitometric analysis is reported in (**H**), where values are means + S.E.M. of three mice per group. * Statistically significant vs. the corresponding values of wild-type mice. Student’s *t*-test, t_4_ = 3.222; *p* = 0.0322.

Immunoblot analysis in the cerebral cortex and cerebellum of wild-type and mGlu5^−/−^ mice showed two bands at 18 and 23 kDa for MBP, and a single band at 100 and 28 kDa for MAG and MOG, respectively (see Figure 1 and Figure 2, and Figure S1A,B). In both regions of the two genotypes, MBP, MAG, and MOG were nearly undetectable at PND 4, and their levels progressively increased from PND 14 to PND 30 (Figure S1A,B). We therefore compared the two genotypes, measuring mRNA levels at PND 4 and PND 7, and both mRNA and protein levels at PND 14, PND 21, and PND 30.

At PND 4 (Figure S1C) and PND 7 (Figure S1D), we found no difference in MAG, MBP, and PDGFRα mRNA levels in the cerebral cortex and cerebellum of the two genotypes. MOG mRNA levels were undetectable at these timepoints. At PND 14, we found a large reduction of MAG, MOG, and MBP mRNA and protein levels in the cerebral cortex of mGlu5^−/−^ mice with respect to their wild-type counterparts (Figure 1A,D). No changes were found in the transcript of PDGFRα (Figure 1A).

At the time of weaning (PND 21), only MBP mRNA levels were still reduced in the cerebral cortex of mGlu5^−/−^ mice, whereas MAG, MOG, and PDGFRα mRNA levels were unchanged (Figure 1B). Protein levels of MAG, MOG, and MBP did not differ between the two genotypes in the cerebral cortex at PND 21 (Figure 1E). At PND 30, there was no difference between wild-type and mGlu5^−/−^ mice in the cortical levels of MAG, MOG, MBP, and PDGFRα mRNA (Figure 1C), whereas MAG and MBP (but not MOG) protein levels were significantly reduced in mGlu5^−/−^ mice (Figure 1F).

In the cerebellum at PND 14, MOG, and MBP mRNA levels were reduced and, interestingly, PDGFRα mRNA levels were significantly increased in mGlu5^−/−^ mice (Figure 2A). MAG, MOG, and MBP protein levels were largely reduced in mGlu5^−/−^ mice (Figure 2D). At PND 21, we found a significant increase in MBP mRNA levels and a reduction in MAG and MBP protein levels in the cerebellum of mGlu5^−/−^ mice. MAG, MOG, and PDGFRα mRNA and MOG protein levels were unchanged (Figure 2B,E). At PND 30, MAG, and MBP mRNA levels were increased in mGlu5^−/−^ mice, whereas levels of the corresponding proteins were reduced. MOG and PDGFRα mRNA and MOG protein levels were unchanged (Figure 2C,F).

Moving from the significant reduction in myelin protein markers observed in mGlu5^−/−^ mice at PND 14, we also performed immunofluorescent staining of MBP at this developmental timepoint. We observed a reduction in MBP immunostaining in the corpus callosum, which, however, did not reach statistical significance (Figure 1G,H), and a significant reduction in the cerebellum of mGlu5^−/−^ mice (Figure 2G,H).

### 3.2. Effect of Early Pharmacological Blockade of mGlu5 Receptors on the Expression of Myelin Protein Markers

**Figure 3:**
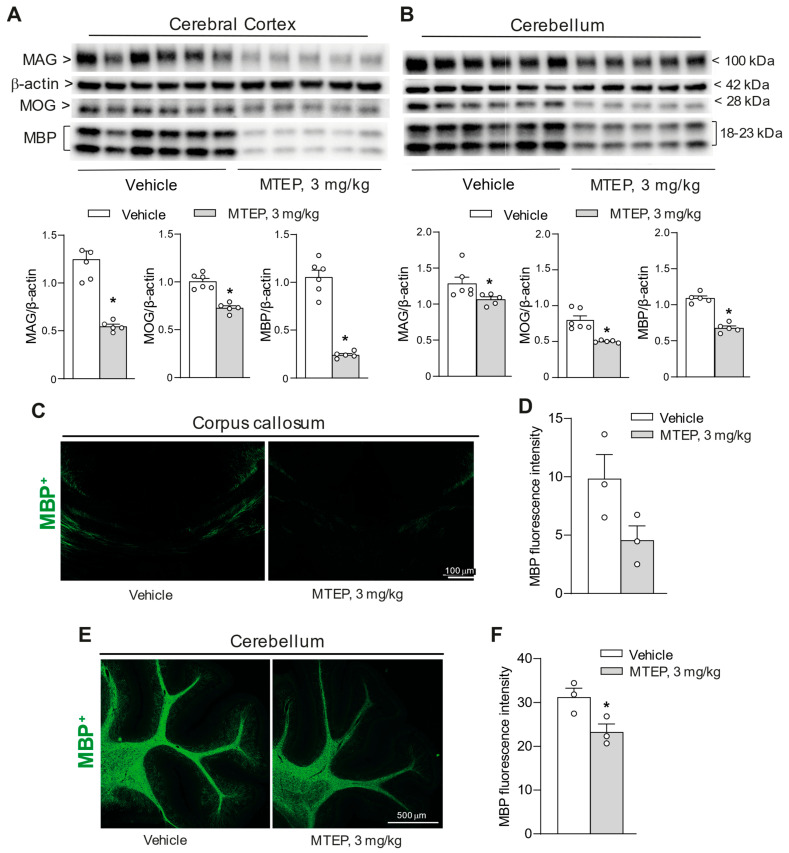
Expression levels of myelin markers in the cerebral cortex and cerebellum of wild-type mice treated with vehicle or MTEP. (**A**,**B**) Expression levels of MAG, MOG, and MBP proteins in the cerebral cortex (**A**) and cerebellum (**B**) after systemic treatment with vehicle or MTEP, 3 mg/kg once a day, from PND 7 to PND 14. Values are means ± S.E.M. of five to six mice per group. * Statistically significant vs. the corresponding values of vehicle-injected mice (Student’s *t*-test for normally distributed values or Mann–Whitney test for non-normally distributed values after Shapiro–Wilk normality test). MAG, t_9_ = 7.196, *p* < 0.0001; MOG, t_9_ = 6.775, *p* < 0.0001; MBP, t_9_ = 10.08, *p* < 0.0001 in (**A**); MAG, U = 2, *p* = 0.0173; MOG, U = 0, *p* = 0.0043; MBP, t_8_ = 9.846, *p* < 0.0001 in (**B**). One statistically significant outlier value was identified by Grubbs’ test in the vehicle group of MBP protein levels in (**B**) and excluded from the analysis. (**C**,**E**) Representative images of immunofluorescent staining of MBP in the medial corpus callosum and in the cerebellum (same region of Figure 2G) of wild-type mice injected i.p. with either vehicle or MTEP. Densitometric analysis is reported in (**D**,**F**), where values are means + SEM of three mice per group. Student’s *t*-test, t_4_ = 2.192; *p* = 0.0935 in (**D**); t_4_ = 2.9; *p* = 0.0441 in (**F**).

To exclude that the delayed maturation of myelin markers observed in mGlu5^−/−^ mice could be due to mechanisms of genetic compensation, we used a pharmacological approach. We treated wild-type mice with the selective mGlu5 receptor NAM MTEP (3 mg/kg, i.p.) once daily from PND 7 to PND 14. This treatment caused a significant reduction in MAG, MOG, and MBP protein levels in the cerebral cortex (Figure 3A) and in the cerebellum (Figure 3B). The reduction of myelin protein markers was confirmed by immunohistochemistry. Wild-type mice treated with MTEP showed a reduction in MBP immunostaining in the corpus callosum, which did not reach statistical significance, and a significant reduction in the cerebellum (Figure 3C–F).

### 3.3. Alterations in Motor Function in mGlu5^−/−^ Mice or in Mice Treated with MTEP

**Figure 4:**
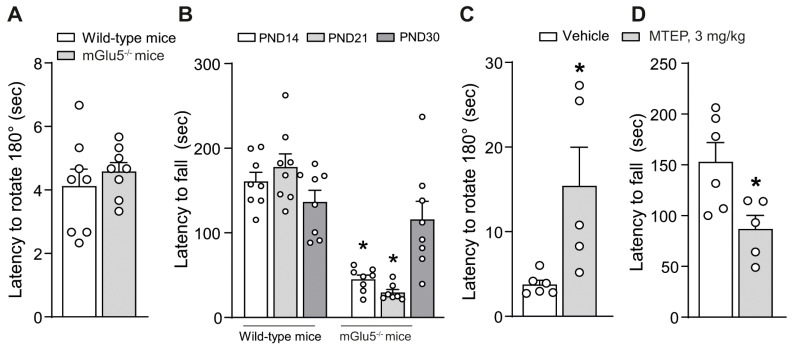
Evaluation of sensorimotor behavior and motor coordination in mGlu5^−/−^ and wild-type mice and in wild-type mice injected with vehicle or MTEP. Values are means ± S.E.M. of five to eight mice per group. * Statistically significant vs. the corresponding values of age-matched wild-type mice (Mixed-effects model for repeated measures + Fisher’s LSD) in (**B**) or vs. the corresponding values of vehicle-injected mice (Student’s *t*-test) in (**A**,**C**,**D**). One statistically significant outlier value was identified by Grubbs’ test in the mGlu5^−/−^ mice group in (**A**) and at PND 21 in (**B**), and excluded from the analysis. t_14_ = 0.7645, *p* = 0.4572 in (**A**); interaction Genotype × Time, F_(2,27)_ = 17.34, p; Genotype, F_(1,14)_ = 46.63; Time, F_(1.684,22.73)_ = 2.859; *p* < 0.0001 wild-type mice vs. mGlu5^−/−^ mice at PND 14 and PND 21 in (**B**); t_9_ = 2.792, *p* = 0.021 in (**C**); t_9_ = 2.75, *p* = 0.0225 in (**D**).

To examine whether the delayed maturation of myelin markers in the cerebral cortex and cerebellum was associated with alterations in the development of motor behavior, we used the negative geotaxis test, which measures the unlearned motor response to gravitation cues and is widely used in developmental studies [27,28], and the rotarod test, which is routinely used for the assessment of motor coordination. Sensorimotor performance in the negative geotaxis test at PND 14 did not differ between mGlu5^−/−^ and wild-type mice (Figure 4A). In contrast, mGlu5^−/−^ mice showed a significant impairment in motor coordination (i.e., a reduction in the latency to fall from the rotarod apparatus) at both PND 14 and PND 21. Motor coordination recovered at PND 30 (Figure 4B). As opposed to mGlu5^−/−^ mice, mice treated with MTEP from PND 7 to PND 14 showed a defect in the ability to turn when placed on an inclined surface in the negative geotaxis test, in addition to a decrease in the latency to fall in the rotarod test with respect to mice treated with saline (Figure 4C,D).

### 3.4. Effect of Pharmacological Blockade of mGlu5 Receptors on Cell Proliferation and Differentiation in Primary Cultures of OPC/Oligodendrocytes

Primary cultures of OPC/oligodendrocytes prepared from the rat cerebral cortex contained < 10% of GFAP^+^ cells and no NeuN^+^ or Iba1^+^ cells (Figure S2A). Cells cultured in proliferation medium for 2 DIV were highly enriched in OPCs, as shown by the expression of PDGFRα and the lack of expression of CNPase by immunoblot analysis. In contrast, cells cultured in differentiation medium for 7 DIV contained mature oligodendrocytes, as shown by the expression of CNPase and the lack of PDGFRα (Figure S2B). We first examined whether a single application of MTEP (1, 3, or 10 μM) could affect OPC proliferation in culture by calculating the ratio of Ki67^+^ Olig2^+^ cells to total Olig2^+^ cells. This treatment did not change Ki67^+^ Olig2^+^/Olig2^+^ cells after 24 or 48 h (Figure S3).

**Figure 5:**
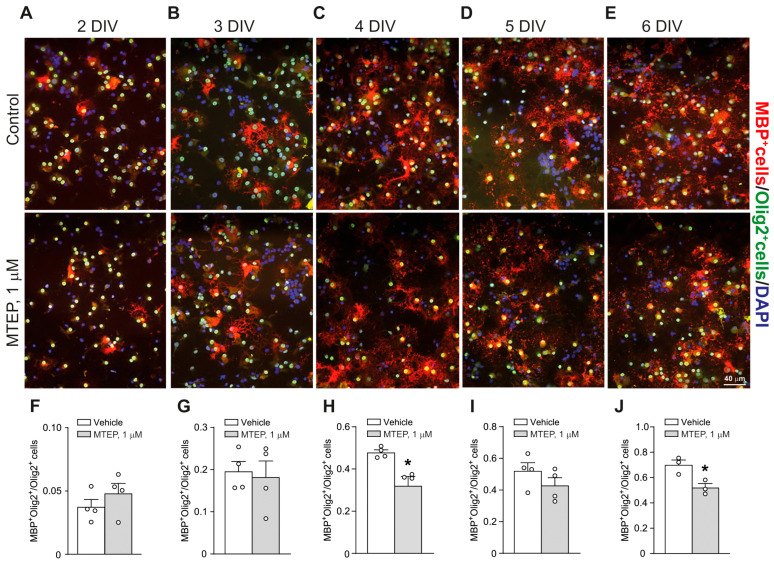
Pharmacological blockade of mGlu5 receptors reduces oligodendrocyte maturation in culture. (**A**–**E**) Representative immunofluorescence staining of MBP (red) and Olig2 (green) in oligodendrocytes cultured in differentiation medium for six days in vitro (DIV) and treated every 24 h with either water (controls) or MTEP (1 µM). Scale bar 40 μm. Cell nuclei were stained with DAPI (blue). (**F**–**J**) The number of MBP^+^ Olig2^+^/Olig2^+^ cells are expressed as means ± S.E.M; *n* = 3–4 independent cultures; each point is the average of values obtained from nine random fields per culture acquired at 20X magnification. * Statistically significant vs. controls (Student’s *t*-test), t_6_ = 10.63, *p* = 0.3289 in (**F**); t_6_ = 0.3068, *p* = 0.7694 in (**G**); t_5_ = 7.285, *p* = 0.0008 in (**H**); t_6_ = 1.291, *p* = 0.2442 in (**I**); t_4_ = 3.566, *p* = 0.0235 in (**J**). One statistically significant outlier value was identified by Grubbs’ test in the MTEP group at 4 DIV in (**H**) and excluded from the analysis.

For the study of cell maturation, we treated cultured oligodendrocytes daily for five consecutive days with either water or MTEP (1 μM) (Figure 5). We assessed the differentiation of primary oligodendrocytes by calculating the ratio of MBP^+^ Olig2^+^ cells to total Olig2^+^ cells. MTEP daily treatment significantly reduced oligodendrocyte maturation at 4 DIV and 6 DIV (Figure 5), without affecting cell viability (see Figure S6). Treatment with MTEP increased the mean cellular MBP fluorescence intensity and reduced the mean area occupied by MBP-fluorescent cells exclusively at 2 DIV (Figure S4). Cell arborisation determined by Sholl analysis was not affected by MTEP at any timepoint (Figure S5).

## 4. Discussion and Conclusions

Data obtained in mGlu5^−/−^ mice or in mice treated with MTEP suggest that endogenous activation of mGlu5 receptors is required for oligodendrocyte maturation and myelin formation during the first two weeks of postnatal life, but, at least in the cerebral cortex, becomes dispensable at later timepoints. In this study, we combined male and female mice from the same litters with no stratification by sex. Although this is a limitation of our study, males and females were combined assuming that sex-related differences are not relevant in the prepubertal age (PND 7–21). In mice, oligodendrocytes are generated from OPCs during postnatal development, and their number peaks in the second week of life, when OPCs rapidly lose their specific markers, such as PDGFRα and NG2 proteoglycans [18,29,30]. Previous studies have shown that mGlu5 receptor expression and function (i.e., agonist-stimulated PI hydrolysis) is substantial between the first and second postnatal weeks and declines afterward [11,12,13,14,16,31]. This strengthens the hypothesis that mGlu5 receptors are linked to oligodendrocyte maturation. However, we did not measure mGlu5 receptor protein levels or signalling in the same samples used for the assessment of myelin markers, and this is a limitation of our study.

mGlu5 receptors are expressed in neurons, astrocytes, and microglia [32], which might regulate myelinogenesis through paracrine mechanisms. For example, insulin-like growth factor-1 and neurotrophin-3, which are produced by many cell types in the CNS, are known to promote oligodendrocyte maturation [33,34]. We used primary cultures of rat OPC/oligodendrocytes devoid of neurons or microglia, and with a small contamination of astrocytes. Pharmacological blockade of mGlu5 receptors with MTEP reduced oligodendrocyte maturation into MBP-expressing cells (i.e., myelin-forming cells) without affecting oligodendrocyte morphology. This suggests that, at least in our culture preparations, it is the activation of mGlu5 receptors expressed by oligodendrocytes that drives oligodendrocyte maturation. A similar scenario was observed in cultured human oligodendrocytes, where pharmacological activation of the mGlu5 receptor with the orthosteric agonist, 2-chloro-5-hydroxyphenylglycine (CHPG), increased the proportion of MBP-expressing oligodendrocytes without affecting cell survival [35]. One of the mechanisms by which mGlu5 receptors might support myelinogenesis is PKC activation, which follows PI hydrolysis and DAG formation [36]. Accordingly, PKC-induced phosphorylation of cAMP response element-binding protein was shown to promote oligodendrocyte maturation [37].

Glutamate binds to mGlu5 receptors with an affinity ranging from 1 to 10 μM [38], and, therefore, mGlu5 receptors present in oligodendrocytes can be activated by the glutamate that spills over from the synapse after being released from nerve terminals [39]. Using functional imaging and optogenetic stimulation in the zebrafish, Braker et al. [24] have shown that neuronal activity drives myelin sheath elongation as a result of mGlu5 receptor activation and the ensuing generation of high-amplitude Ca^2+^ transients in myelin. Thus, the mGlu5 receptor can be the linking bridge between neuronal activity and myelination by oligodendrocytes [24].

The temporal coincidence between the developmental peak of mGlu5 receptor expression and function and myelination in the early postnatal life suggests that mGlu5 receptors might be involved in the pathophysiology of neurodevelopmental disorders associated with myelin dysfunction [1]. In mice treated with MTEP from PND 7 to PND 14, the abnormal myelinogenesis in the cerebral cortex and cerebellum was associated with a substantial behavioral impairment in the negative geotaxis and rotarod tests, which were used for the assessment of sensorimotor function and motor coordination, respectively. mGlu5^−/−^ mice also showed a significant deficit in the rotarod test, but only a trend to a deficit in the negative geotaxis test, suggesting that the sensorimotor deficit could have been rescued by compensatory mechanisms occurring in constitutive mGlu5^−/−^ mice. The limited extent or timing in mGlu5 receptor blockade with MTEP as compared to the absence of mGlu5 receptors in mGlu5^−/−^ mice may also contribute to the different performance in the negative geotaxis test. These data suggest a potential link between mGlu5 receptors, myelin formation, and the behavioral phenotype, raising the possibility that drugs that enhance the activity of mGlu5 receptors may have therapeutic value in neurodevelopmental disorders. In a rat model of periventricular leukomalacia [23], myelin loss was partially rescued by systemic treatment with (1S,3R)-1-aminocyclopentane-1,3-dicarboxylic acid, an orthosteric agonist of mGlu1, −2, −3, and −5 receptor subtypes [38].

In both rodents and humans, OPCs are also present in the adult brain and promote remyelination after injury [9,20,40]. Evidence suggests that mechanisms regulating myelinogenesis during development are conserved in myelin repair in the adult life (the “recapitulation hypothesis” of myelin regeneration) [41,42]. It will be interesting to examine whether myelin regeneration after injury is under the control of mGlu5 receptors as we have found during early postnatal development. Interestingly, mGlu5 receptors are re-expressed in cerebellar Purkinje cells under conditions of inflammatory demyelination, i.e., in mice developing experimental autoimmune encephalomyelitis, or in postmortem tissue of individuals affected by multiple sclerosis [43]. The hypothesis that mGlu5 receptors are involved in myelin regeneration after injury warrants further investigation.

## Data Availability

Data are available on the Neuromed Repository (access starting on 21 July 2026 (https://repository.neuromed.it/index.php/s/enf4yLZoDHEcTcJ)).

## Supplementary Materials

The following supporting information can be downloaded at: https://www.mdpi.com/article/10.3390/cells15141311/s1. Figure S1: Postnatal expression profile of structural components of myelin in the developing cerebral cortex and cerebellum of wild-type and mGlu5^−/−^ mice; Figure S2: Cell purity assessed by immunofluorescence using lineage-specific markers; Figure S3: Pharmacological blockade of mGlu5 receptors does not affect OPC proliferation in culture; Figure S4: Time-dependent effect of mGlu5 receptor blockade on oligodendrocyte morphology; Figure S5: Pharmacological blockade of mGlu5 receptors does not affect the morphological complexity of oligodendrocytes in culture; Figure S6: MTEP does not induce cytotoxicity in oligodendrocyte cultures.
