# Supplementary material for: Endogenous Activation of mGlu5 Metabotropic Glutamate Receptors Is Required for Oligodendrocyte Maturation and Myelinogenesis in Mice
Source: Cells. 2026 Jul 22;15(14):1311. doi: 10.3390/cells15141311 (PMC13406444; doi:10.3390/cells15141311)
Supplement: Supplementary file 1 [file cells-15-01311-s001.zip › cells-4307524-supplementary.pdf]

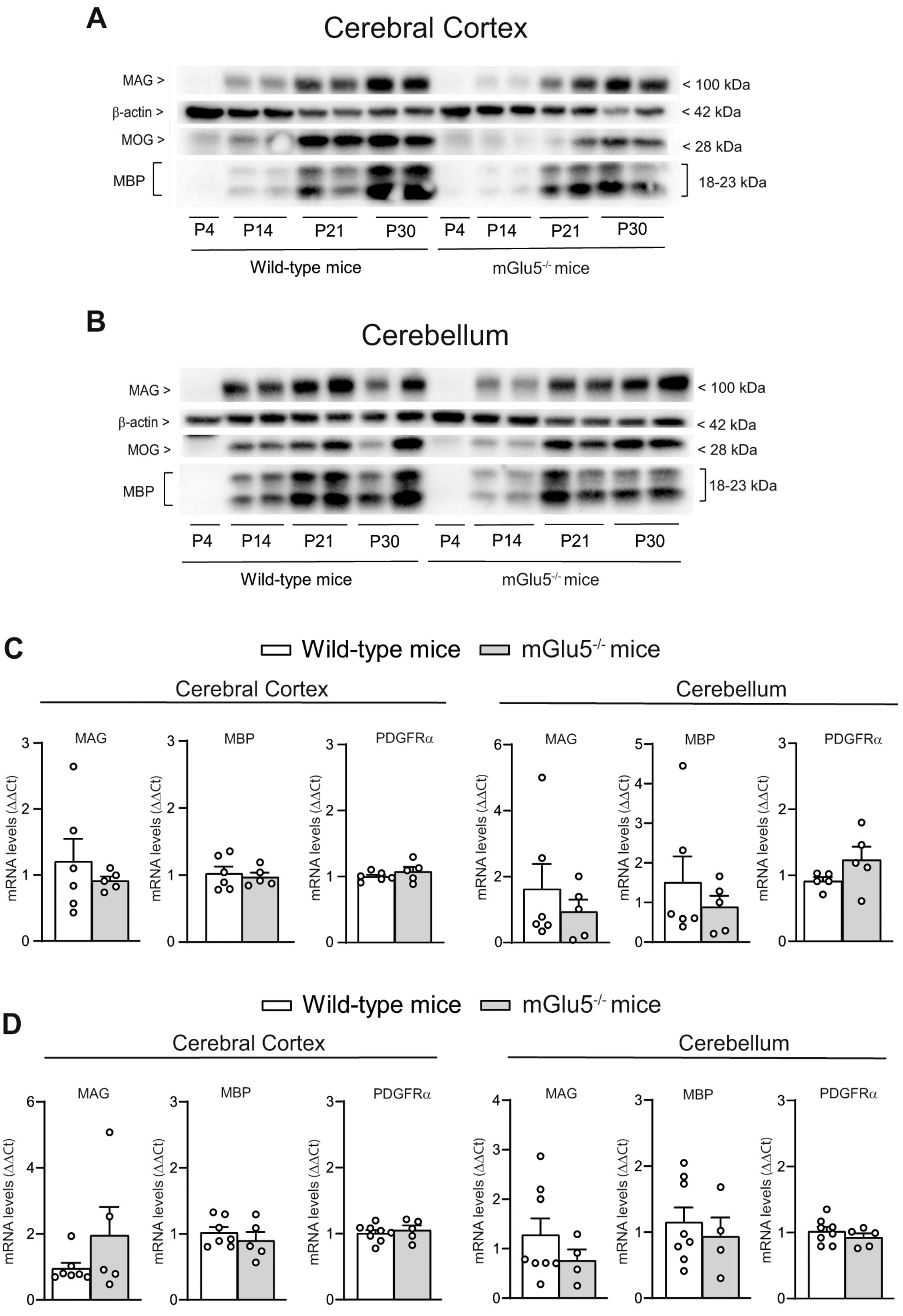

A
Cerebral Cortex
MAG >
β-actin >
MOG >
MBP
< 100 kDa
< 42 kDa
< 28 kDa
18-23 kDa
P4
P14
P21
P30
Wild-type mice
mGlu5-/- mice
B
Cerebellum
C
Wild-type mice
mGlu5-/- mice
Cerebral Cortex
Cerebellum
MAG
MBP
PDGFRα
mRNA levels (ΔΔCt)
D

Figure S1

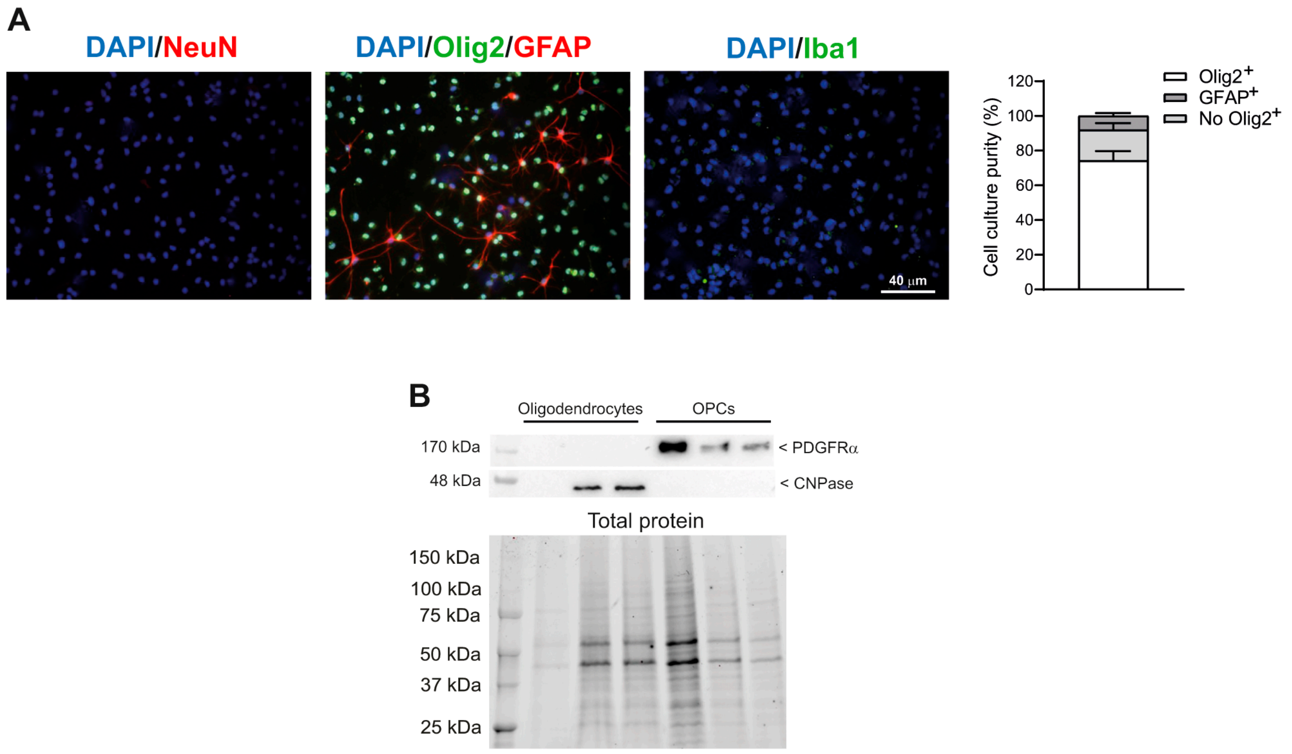
A
DAPI/NeuN
DAPI/Olig2/GFAP
DAPI/Iba1
40 µm
Olig2+
GFAP+
No Olig2+
Cell culture purity (%)
120
100
80
60
40
20
0
B
Oligodendrocytes
OPCs
170 kDa
48 kDa
< PDGFRα
< CNPase
Total protein
150 kDa
100 kDa
75 kDa
50 kDa
37 kDa
25 kDa

Figure S2

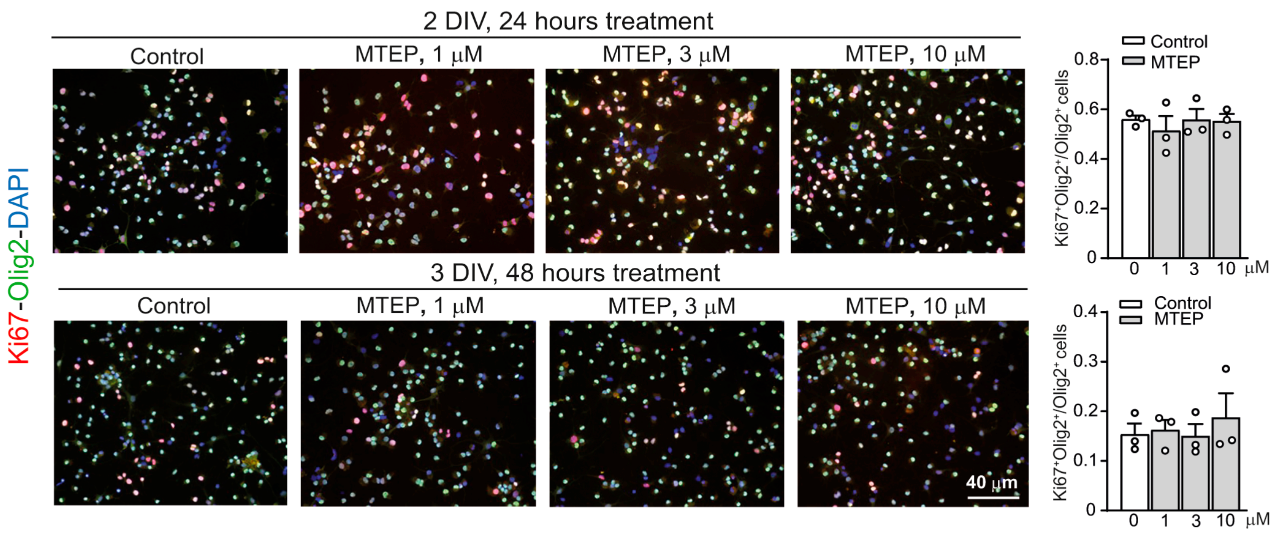
2 DIV, 24 hours treatment
Control
MTEP, 1 μM
MTEP, 3 μM
MTEP, 10 μM
3 DIV, 48 hours treatment
Control
MTEP, 1 μM
MTEP, 3 μM
MTEP, 10 μM
40 μm
Ki67-Olig2-DAPI
Control
MTEP
Ki67+Olig2+/Olig2+ cells
0.8
0.6
0.4
0.2
0
0 1 3 10 μM
Control
MTEP
Ki67+Olig2+/Olig2+ cells
0.4
0.3
0.2
0.1
0
0 1 3 10 μM

Figure S3

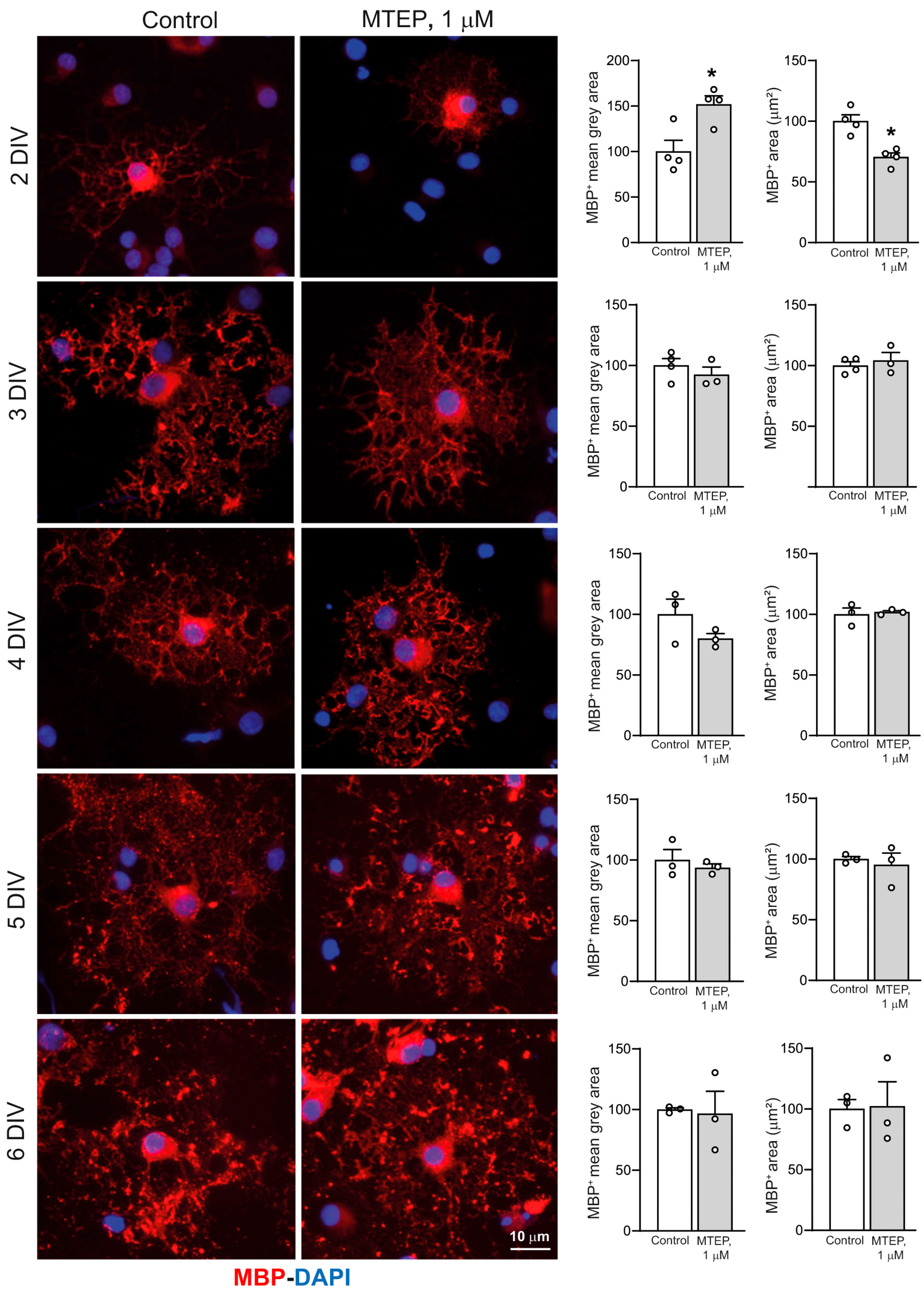

Control
MTEP, 1 μM
2 DIV
3 DIV
4 DIV
5 DIV
6 DIV
10 μm
MBP-DAPI
MBP⁺ mean grey area
MBP⁺ area (μm²)
Control
MTEP, 1 μM

Figure S4

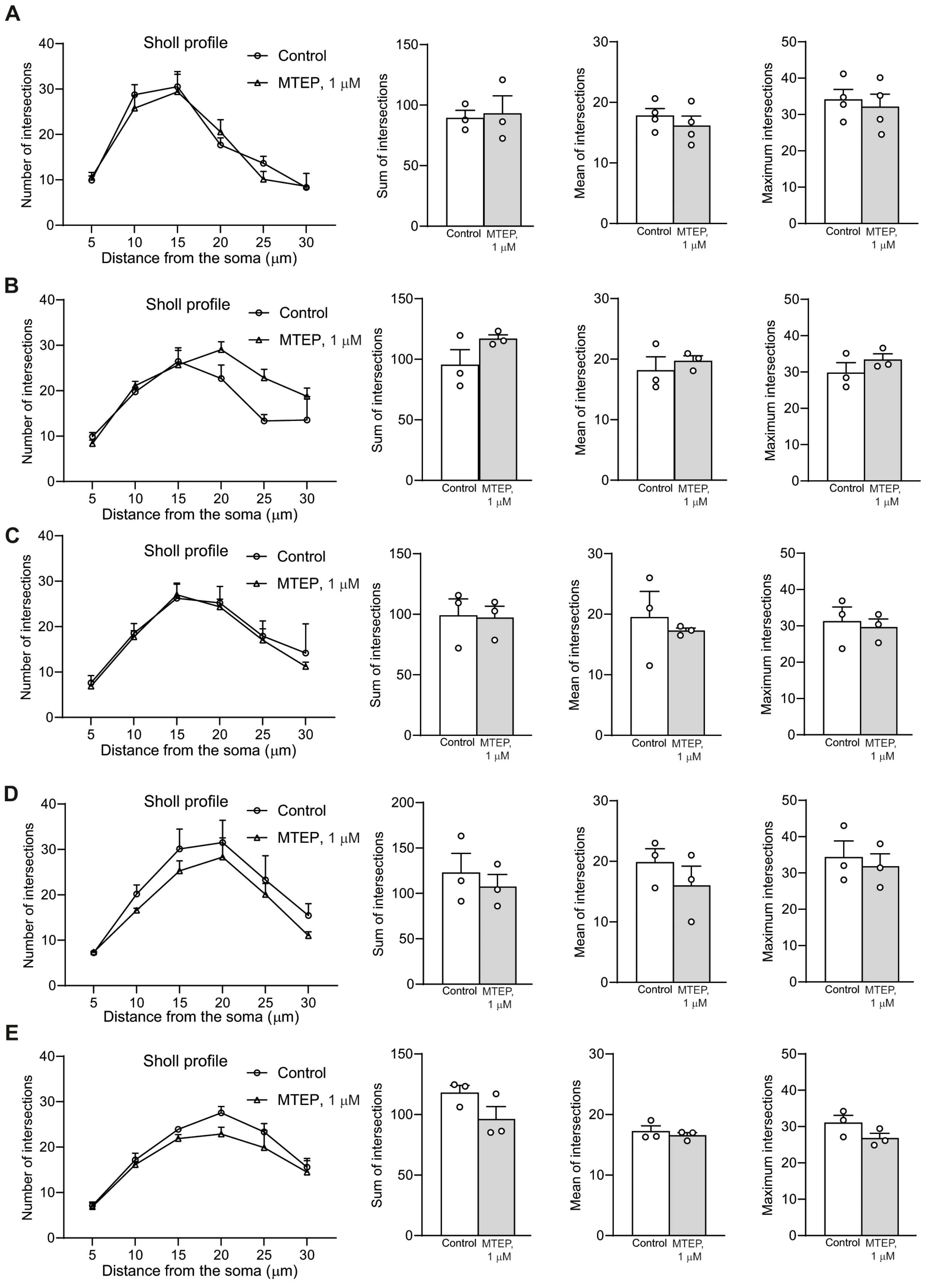
A
Sholl profile
Control
MTEP, 1 μM
Number of intersections
Distance from the soma (μm)
Sum of intersections
Mean of intersections
Maximum intersections
Control
MTEP, 1 μM
B
Sholl profile
Control
MTEP, 1 μM
Number of intersections
Distance from the soma (μm)
Sum of intersections
Mean of intersections
Maximum intersections
C
Sholl profile
Control
MTEP, 1 μM
Number of intersections
Distance from the soma (μm)
Sum of intersections
Mean of intersections
Maximum intersections
D
Sholl profile
Control
MTEP, 1 μM
Number of intersections
Distance from the soma (μm)
Sum of intersections
Mean of intersections
Maximum intersections
E
Sholl profile
Control
MTEP, 1 μM
Number of intersections
Distance from the soma (μm)
Sum of intersections
Mean of intersections
Maximum intersections

Figure S5

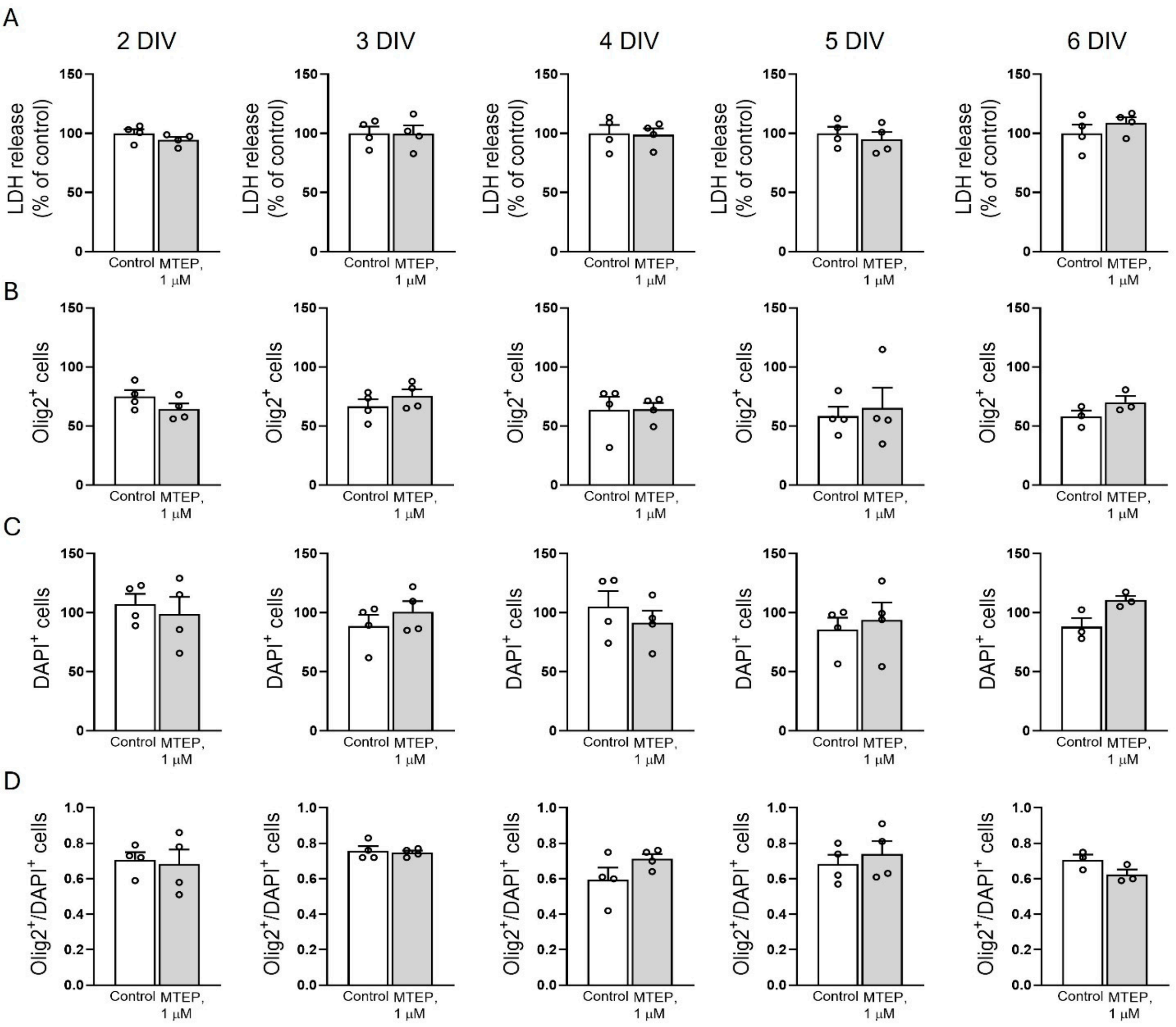
A
2 DIV
3 DIV
4 DIV
5 DIV
6 DIV
LDH release (% of control)
B
Olig2+ cells
C
DAPI+ cells
D
Olig2+/DAPI+ cells
Control
MTEP, 1 μM

Figure S6

Supplementary Figure legends

Figure S1. Postnatal expression profile of structural components of myelin in the developing cerebral cortex and cerebellum of wild-type and mGlu5$^{-/-}$ mice.

(**A,B**) Representative immunoblot analysis in the cerebral cortex (**A**) and cerebellum (**B**) of wild-type and mGlu5$^{-/-}$ mice. (**C,D**) Transcript levels of myelin and oligodendrocytes markers in the cerebral cortex and cerebellum of wild-type and mGlu5$^{-/-}$ mice at PND 4 (**C**) and PND 7 (**D**).

Figure S2. Cell purity assessed by immunofluorescence using lineage-specific markers: NeuN for neurons, Iba1 for microglia, GFAP for astrocytes, and Olig2 for oligodendrocyte lineage cells.

(**A**) Representative micrographs and corresponding quantification. Images were acquired at 20× magnification using a Nikon i80 microscope. Cells were manually counted in nine randomly selected fields per preparation using Fiji software to determine the proportion of marker-positive cells. Quantitative analysis showed a culture purity of 92%, with 8% of cells identified as GFAP-positive astrocytes. Values are expressed as means ± S.E.M. of n = 3 independent cultures. (**B**) Representative immunoblot analysis of cells cultured in proliferation medium for 2 days *in vitro* (2 DIV) and differentiated oligodendrocytes at 7 DIV. Membranes were probed with anti-CNPase and anti-PDGFRα antibodies to assess oligodendrocyte differentiation status.

Figure S3. Pharmacological blockade of mGlu5 receptors does not affect OPC proliferation in culture.

Representative immunofluorescence staining of Ki67 (red) and Olig2 (green) in OPCs cultured in proliferation medium and treated for 24 hours, fixed at 2 days *in vitro* (DIV), or 48 hours, fixed at 3 DIV, with either water or MTEP (1, 3 or 10 μM). Scale bar 40 μm. The number of Ki67^{+} Olig2^{+}/Olig2^{+}

cells is expressed as means ± S.E.M (n = 3 independent cultures; each point is the average of values obtained from 9 random fields per culture acquired at 20X magnification).

Figure S4. Time-dependent effect of mGlu5 receptor blockade on oligodendrocyte morphology.

Representative photomicrographs of MBP^+ cells cultured in differentiation medium for 6 days *in vitro* (DIV) and treated every 24 hours with either water or MTEP (1μM). Scale bar 10 μm. Cell nuclei were stained with DAPI (blue). MBP expression is expressed as the mean grey value, whereas the area of MBP^+ signal is expressed in μm^2. Values are expressed as means ± S.E.M of n = 3-4 independent cultures; 20 single cells were acquired at 40X magnification for each coverslip. *Statistically significant vs. controls (Student's t-test), 2 DIV MBP^+ mean grey area $t_6 = 3.319$, $p = 0.016$; 2 DIV MBP^+ area $t_6 = 4.596$, $p = 0.037$.

Figure S5. Pharmacological blockade of mGlu5 receptors does not affect the morphological complexity of oligodendrocytes in culture.

(**A-E**) Number of intersections at different distances from cell soma and cumulative results obtained from Sholl analysis performed with Fiji on MBP^+ oligodendrocytes cultured in differentiation medium for 6 days *in vitro* (DIV), 2 DIV in **A**, 3 DIV in **B**, 4 DIV in **C**, and 5 DIV in **E**. Cells were treated every 24 hours with either water or MTEP (1 μM) (see photomicrographs in Fig. S5). Sholl profile: concentric circles were juxtaposed on each MBP^+ cell starting from 10 μm far from the cell soma and spaced 5 μm apart. Cumulative results: sum of intersections is the number of intersections counted in each circle; mean of intersections is the ratio sum of intersections /number of circles; maximum number of intersections is the highest number of processes/branches counted in each MBP^+ cell. Values are expressed as means ± S.E.M of n = 3 independent cultures; 20 single cells were acquired at 40X magnification for each coverslip.

Figure S6. MTEP does not induce cytotoxicity in oligodendrocyte cultures.

(**A**) LDH release assay performed on culture supernatants from oligodendrocytes. (**B**) Quantification of Olig2-positive cells at the indicated time points. (**C**) Quantification of DAPI-positive nuclei at the indicated time points. (**D**) Quantification of Olig2/DAPI-positive cells at the indicated time points.

In **A**, **B**, **C**, and **D**, cells were cultured for 6 days in vitro (DIV) in differentiation medium and treated every 24 h with either water or MTEP (1 μM), beginning 24 h after seeding. Values are expressed as means ± S.E.M. of n = 3–4 independent cultures; each point represents the average of values obtained from 9 random fields per culture acquired at 20× magnification. No significant differences were observed between groups.
